# Structure Influences Case Processing: Electrophysiological Insights from Hindi Light Verb Constructions

**DOI:** 10.3390/brainsci16020176

**Published:** 2026-01-31

**Authors:** Anna Merin Mathew, R. Muralikrishnan, Mahima Gulati, Kamal Kumar Choudhary

**Affiliations:** 1 Indian Institute of Technology Ropar, Rupnagar 140001, Punjab, India; 2017hsz0001@iitrpr.ac.in (A.M.M.); kamal@iitrpr.ac.in (K.K.C.); 2 Max Planck Institute for Empirical Aesthetics, Grüneburgweg 14, 60322 Frankfurt am Main, Germany; 3 New Delhi Institute of Management, Delhi 110062, India; mahima.gulati@ndimdelhi.org

**Keywords:** case, light verbs, aspect, N400, P600, Hindi

## Abstract

**Background:** Case marking serves as a crucial cue in sentence processing, enabling the prediction of upcoming arguments, thematic roles, and event structure. Cross-linguistic studies have revealed language-specific variations in case processing, with differences observed between nominative–accusative and ergative languages, albeit with limited data from the latter. **Objective:** To this end, we investigated case processing in Hindi compound light verb constructions, leveraging its split-ergative system. **Methods:** An ERP study was conducted with twenty-four native Hindi speakers, wherein the subject case (ergative or nominative) either matched or mismatched with the aspect marking on the light verb (perfective or imperfective). **Results:** The results revealed distinct ERP effects depending upon the subject case: a P600 effect for ergative case violations at the imperfective light verb and a biphasic N400-P600 effect for nominative case violations at the perfective light verb. **Conclusions:** These findings suggest underlying neurophysiological differences in the processing of ergative versus nominative case alignment within light verb structures. Moving forward, a closer examination of structure-specific neurophysiological variation can help bridge the gap between typological distributions and their neural underpinnings.

## 1. Introduction

Language comprehension relies on multiple linguistic cues whose relative salience is shaped by cross-linguistic variation in typological features. These cues differ in their reliability and availability across languages, and native speakers weigh them according to their predictive value for interpreting sentence structure [1,2,3,4].

Among these available cues, case marking has emerged as a particularly informative cue, guiding the real-time mapping of syntactic roles and thematic relations [1]. Cross-linguistic research over the past few decades has investigated whether case functions as an equally prominent processing cue across languages. These studies have revealed that case markers encode core relational information, facilitating argument disambiguation, thematic assignment, and the prediction of upcoming arguments and the verb, along with their associated features. In addition, the findings indicate that when case marking conflicts with other cues, comprehenders dynamically negotiate competing sources of information during real-time processing [5,6,7,8,9] (refer to the cue validity framework, in which sentence processing is viewed as a dynamic competition among cues, while the conflict reliability model further accounts for processing difficulty by showing that comprehension is most disrupted when highly reliable cues provide conflicting information [4,10]).

Recently, psycholinguistic research on typologically diverse languages gained momentum [11,12,13,14], where the investigation of ergativity has sparked intense debate, as it offers a rich terrain for cross-linguistic comparison, highlighting both variation and universals in languages [15,16,17,18,19,20,21,22,23,24,25,26]. In this vein, electrophysiological evidence on the processing of case and case-based violations from ergative language has shown different ERP signatures, depicting variation even within ergative language types. In Basque, an auditory ERP study reported a positivity at the second ergative-marked argument in response to double ergative case violations in SOV sentences. The positivity effect was interpreted as reflecting the detection of case-based violations [27], aligning with ERP responses to case violations in German [6,28], but it contrasts with the N400 effects reported for similar violations in Hindi [29]. Another ERP study [30] showed that native Basque speakers exhibit a biphasic N400-P600 pattern for ergative violations on sentence end subjects (OVS), whereas Spanish-speaking non-natives show only an N400. The N400 reflected difficulty in thematic role assignment following the absence of ergative marking, while the P600 (observed only in native Basque speakers) was taken to reflect error detection and syntactic reanalysis. The non-natives do not find the violations as a strict error, as they rely more on semantic cues like animacy (from Spanish) than on structural case information.

In a split-ergative language, Hindi, an auditory ERP study reported distinct processing for case and aspect violations: an N400-P600 for ergative–imperfective violations with an increased amplitude, whereas nominative–perfective violations elicited an N400 at the clause-final verb in SOV sentences [29]. The N400 was interpreted as reflecting the violation of an interpretively relevant case-based constraint, with ergative violations imposing greater semantic or interpretive difficulty. The P600 effect observed for ergative case violations was attributed to the non-default nature of ergative marking in Hindi, which is restricted to specific contexts (perfective aspect with transitive verbs). Converging evidence comes from a visual ERP study from another split-ergative language, Punjabi, which reported a positivity for both the ergative and nominative case and aspect violations, with typographical disparities. Despite differences in scalp distribution, the P600 effect showed comparable sensitivity to ill-formed constructions across subject case types [31]. Another Hindi ERP study reported a right anterior negativity (RAN)-P600 for mismatches between ergative case and verbal morphology in SOV sentences. The RAN was taken to index the processing cost of resolving a morphosyntactic dependency between an ergative argument and its expected clause-final verb, while the P600 again reflected the detection of an ill-formed case configuration [32]. Together, these findings from Hindi [29,32] suggest that the ergative case is a strong syntactic cue for sentence comprehension, while Punjabi reports that the reliance on case as a cue may vary depending on the language [31]. Collectively, these ERP findings suggest that the processing of case may be sensitive to both language and construction-specific grammar.

The above-mentioned studies have primarily used simple transitives to denote event structures, where the effect of case processing was studied either on the argument or on single transitive verbs. However, we know that languages of the world can use different kinds of sentences, such as a complex predicate structure, to convey the same event. The languages we speak and how we structure events influence our thoughts and understanding of the world [33]. In this regard, we question whether case would be processed similarly if we utilize a novel structural configuration, i.e., light verb structures, to understand its comprehension. 

There exists a limited body of experimental research examining the complexity of the semantic and/or syntactic properties of light verb structures, using various methodologies. These studies have shown us that light verb structures are processed differently than other types of non-light structures, resulting in distinct neural responses, such as a frontally focused late sustained negativity [34], and varying processing costs across languages [35,36,37,38]. However, most of these studies mainly contrasted conjunct light verb structures with different types of non-light predicate structures to investigate the processing costs of argument sharing and event representation in light verb predicates. Some of these studies used Hindi conjunct LVCs to study prediction strength, event interpretation, and memory retrieval. Such studies have reported that readers form strong expectations about upcoming predicates, facilitating processing even in LVC contexts [39]. Light verbs in perfective constructions can signal event completion, though interpretation remains context-sensitive [40], and complex LVC predicates interact with memory-based agreement processes, where distractor nouns can trigger attraction errors [41]. However, thus far, none of the studies mentioned above have directly examined how case and case-based violations are processed, nor have they questioned whether case functions as an equivalent processing cue across different structures. This becomes particularly interesting when questioned from the position of a second/light verb in a light verb construction whose grammatical features, along with case patterns, jointly shape sentence comprehension.

Notably, an exception to the aforementioned pattern is provided by an ERP study employing Hindi compound light verb structures to investigate the processing of ergative and nominative case to their associated transitivity-based violations [42]. Drawing on the experimental evidence coming from studies on processing case and case-based violation as well as studies comparing light verb structures to other predicate types, this study contributes further evidence to the investigation of case processing. The study asks whether case processing would vary when the transitivity of the light verb differs in compound light verb structures. It kept the aspect-based split-ergativity rules intact while manipulating the transitivity-based split-ergative system in Hindi. The interplay between the subject initial case (ergative versus nominative case) and the transitivity of the light verb/V2 (intransitive versus transitive light verb) revealed completely distinct ERP patterns on the light verb. The results showed an N400 effect for the ergative case-intransitive light verb violation in comparison to ergative case-transitive light verb correct conditions. Further, a P600 was evoked for the nominative case-transitive light verb violation in comparison to the nominative case-intransitive light verb correct conditions. The authors suggested that this was indicative of neurophysiological distinction in the processing of the split-ergative case system when regulated by the transitivity of the light verb. All these electrophysiological findings together suggest considerable variation in case processing within ergative languages. In lieu of these findings, there is a need for cross-linguistic research to investigate the computation of structure-specific variations in case processing.

In the following sections, we discuss the case alignment system and light verb structures in Hindi (Section 1.1) and outline the motivation for the present study along with our hypothesis (Section 1.2). The remainder of the paper describes the methodology, including participant information, materials, experimental procedures, EEG data recording and preprocessing, and statistical analyses (Section 2). This is followed by the behavioral and ERP results (Section 3), leading to a comprehensive discussion (Section 4). Finally, we summarize the main findings and outline future research directions (Section 5).

### 1.1. Hindi Case System and Compound Light Verb Constructions

Hindi is an Indo-Aryan language that has a flexible word order pattern with SOV as the default pattern. The language has a rich case-marking morphology wherein nominals bearing clitics/postpositions are marked for case. The case marking system in Hindi exhibits a split-ergative system conditioned by aspect/tense and transitivity [16,43], where ergative–absolutive and nominative–accusative case alignment patterns coexist. The transitivity-driven case alignment system in Hindi was thoroughly discussed and experimentally studied in [42]. In the present study, we will focus on the aspect-based split-ergativity system, which governs case alignment rules, distinguishing between perfective and imperfective aspects [15,44,45,46,47]. This framework provides a unique context for investigating case processing in light verb structures.

The Hindi language, like most South Asian languages, exhibits quite vigorously the phenomenon of complex predicate structures, which are widely spread and incorporate a variety of different predicate types. The complex predicates are theoretically thoroughly debated upon but experimentally less explored with respect to their nature, subtypes, and the semantic properties and grammatical functions of each unit in the composition [48,49,50,51,52,53,54,55,56], among many others.

Light verb constructions (LVCs) are one such type of complex predicate structures, commonly analyzed within the universal predicational framework of argument–event co-composition, wherein LVCs are an amalgamation of their semantic and syntactic composition. In an LVC, two or more predicational elements (such as nouns, verbs, and adjectives), which function as a single element, are mapped onto a monocausal syntactic structure [43,52,53]. A central property of this composition is argument sharing, whereby the light verb does not introduce an independent set of arguments but shares its arguments, typically the external argument, with the other element, allowing the construction to function as a single predicate [57]. This distributed predication and shared argument structure has been argued to hold cross-linguistically, including in Hindi and other languages, with productive light verb systems [52,53,54]. Additionally, this forms the theoretical basis for analyses of both the grammatical behavior and processing characteristics of light verb constructions (several theoretical approaches that have tried to decipher the complexity of light verb structures, such as the separate entry approach by [58], within the Construction Grammar framework, [59], within the Minimalist Program, the underspecification approach and parallel architecture framework by [36,60,61,62], etc., to name a few.)

Consider the following examples in Hindi, wherein 1(a) depicts ergative case assignment governed by the main verb in a simple predicate structure, while 1(b) represents ergative case assignment in compound light verb construction.
1(a)Raam-negharkhareed-aahai
Ram.3SG.M-ERGhouse.3SG.M.NOMbuy-PFV.3SG.MAux.SG.PRS
‘Ram has bought a house’

1(b)Raam-negharkhareedli-yaahai
Ram.3SG.M-ERGhouse.3SG.M.NOMbuytake-PFV.3SG.M.LVAux.SG.PRS
‘Ram bought a house’

In 1(a), a single transitive main verb ‘khareedaa’ (meaning ‘bought’) carries the required grammatical markers to establish for aspect/tense, agreement (person/number/gender), and case assignment relations. Additionally, the main verb bears the semantic weight of event structure, as the meaning of the sentence, which denotes the notion ‘of having bought a house’, comes from ‘khareedaa’. The perfective aspect of the transitive verb, along with the presence of the overtly marked ergative case in Hindi, presents events as completed wholes, implying agentive and volitional subjects [15]. Most of the previous ERP studies from split-ergative languages have examined the processing of case from the position of the main verb (Hindi: [29,32]; Punjabi: [31]). However, this position often represents a processing bottleneck, as it integrates multiple semantic and syntactic operations crucial for real-time sentence comprehension.

On the other hand, in 1(b), we highlight a specific type of transitive light verb structure known as the compound light verb constructions, wherein two predicating constituents (V1 + V2) combine to denote a single argument structure. There is a bifurcation of semantic and syntactic functions onto these two verbs, such that each element has its own role to perform to establish a joint predication. The first/polar verb (V1) ‘khareed’ (meaning ‘buy/purchase’) is the main transitive verb in the stem/root form. This first part of the predicate structure contributes most of the lexical meaning, and its semantic transitivity determines the valency of the event structure. On the other end, the second/light verb (V2) ‘liyaa’ (meaning ‘take’) has been known by many names, an ‘explicator verb’ [63], ‘light verb’ [64], vector verbs [65], intensifying verbs, operator verbs, compound auxiliaries, etc., [52,53]. The light verb (V2) carries grammatical markers, such as tense, aspect, case, and agreement information, which are essential for establishing the grammatical structure. The light verb in 1(b) is marked transitive and perfective, assigning ergative case to the subject argument [43,52,53,54] and thereby influencing the sentence interpretation. Sentence 1(b) demonstrates how the ergative case assignment is governed by the light verb/V2 ‘liyaa’ in the perfective aspect, denoting a completed event. In Hindi, the perfectivity and transitivity pattern of the light verb is of the core entities governing the split-ergativity nature of the language [16,17,19,66,67,68].

The light verb historically is said to have undergone a grammaticalization process and is considered a semantically bleached/underspecified verb, hence ‘light’, as it contributes minimally to the predicate’s core meaning [54,69,70]. The meaning of the complex predicate structure ‘khareed liyaa’ (meaning ‘bought a house’) is mostly derived from ‘khareed’ V1 (buy) compared to the light verb ‘liyaa V2 (take). However, the light verbs are not semantically empty; they do have some extra-linguistic information that they carry; for example, intensity, violence, stubbornness, reluctance, regret, forethought, and thoroughness [71,72], forcefulness, suddenness, volitionality [43,72], agentivity, benefaction, directionality [43,48,72], and features such as finality, definiteness, manner of the action, attitude/intention of the speaker, a sense of negative value, etc. [48,52,63]. Exceptions to these include some specific complex predicate structures, like conjunct light verb structures and permissives, which exhibit many distinct case/agreement alignment patterns [19,66,68,71,72]. Finally, both the elements in a compound light verb structure yield a unified event representation through their interaction [43,73].

In contrast, sentence 1(c) is another type of a transitive compound light verb construction wherein the nominative case assignment is governed by an imperfective light verb. The imperfective aspect focuses on unfolding actions with uncertain completion, often associated with less agentive subjects.
1(c)*Raam**ghar**khareed**le-taa**hai*
Ram.3SG.M.NOMhouse.3SG.M.NOMbuytake-IMPF.3SG.M.LVAux.SG.PRS
‘Ram buys a house’

Further research on the aspectual properties of Hindi verbs has revealed that simple and compound light verbs differ in how they encode event interpretation. Compound verbs, formed with light verbs like ‘liyaa’ or ‘letaa’, signify attainment of natural endpoints, emphasizing completion or change in state, whereas simple verbs allow for more arbitrary endpoints [45,46,47]. The aspectual distinction is not entirely semantic but interacts with the verb’s syntactic profile, altering its transitivity, which in turn governs the case assignment patterns. It is important to first tease apart and separately examine the semantic as well as syntactic complexity of different types of predicate structures to make better claims about their processing mechanism. For this reason, light verb construction, in contrast to simple transitive, offers a comparatively better division of labor and a novel sentential configuration for examination. Thus, to begin with, studying Hindi’s split-ergative system in a compound light verb structure would provide insights into how light verbs introduce subtle nuances into the event structure, shaping the reader’s understanding of both the case assignment and the degree of completion, ease, or intentionality of an action [19,53,66,68].

In view of this as our typological grounding, the present study investigated the processing of ergative and nominative case assignment in Hindi compound light verb constructions.

### 1.2. The Present Study

The present visual ERP study investigated the processing of ergative and nominative case assignment in Hindi compound light verb constructions to examine whether they are neurocognitively similar, evoking similar ERP signatures during real-time comprehension. For this purpose, the aspect governed split-ergative case alignment system in Hindi provides us the opportunity to analyze how the predication held by the subject case markers for the upcoming sentence interpretation is altered when the grammaticality of case assignment is governed by the aspectual properties of the light verb. Additionally, the aim was also to determine whether a varied structural configuration would impact the processing of the two types of subject case assignment, especially when examined within a complex predicate structure.

To accomplish this, an event-related potential study was employed with a 2 × 2 factorial design manipulating the subject case (ergative or nominative case) and aspect markers on the light verb (perfective or imperfective aspect) in Hindi compound light verb constructions. This resulted in four conditions (Table 1): nominative–imperfective controls (NOM-IMPF) were compared to ergative–imperfective violation (*ERG-IMPF) conditions, and ergative–perfective controls (ERG-PERF) were compared to nominative–perfective violation (*NOM-PERF) conditions. The experimental conditions were structured in the following order: adverb—subject (NP1 with ERG or NOM case)—object (NP2 with default NOM case)—main verb (V1; root form)—light verb (V2 with IMPF or PERF aspect)—auxiliary. For example, aaj (adverb: today) Lekhak-ne (NP1: writer.3SG.M-ERG) patr (NP2: letter.3SG.M.NOM) padh (V1: read) li-yaa (V2: take.PERF.M.3SG.LV) hai (Aux: SG.PRS) ‘Today, the writer has read the letter’.

In the control condition, ergative–perfective (ERG-PERF), an ergative case-marked subject argument matched with the following transitive second verb/light verb in the perfective aspect. Thereby, accurately implementing the ergative subject case assignment. For the control condition, nominative–imperfective (NOM-IMPF), a nominative case-marked subject argument matched with the following transitive second verb/light verb in the imperfective aspect. Thus, correctly displaying the nominative subject case assignment. In contrast, the violation conditions, nominative–perfective (*NOM-PERF) and ergative–imperfective (*ERG-IMPF), demonstrate a subject case and aspect assignment mismatch, thereby violating the aspect-governed split-ergativity case system in Hindi. In the critical violation condition, nominative–perfective (*NOM-PERF), the nominative case-marked subject argument was followed by a transitive second verb/light verb in the imperfective aspect. This leads to a nominative case violation, which ensues from an erroneous usage of the nominative case with the transitive light verb in the perfective aspect. In the ergative–imperfective (*ERG-IMPF) violation condition, the ergative case-marked subject argument was followed by a transitive second verb/light verb in the perfective aspect. Henceforward, it causes an ergative case assignment violation stemming from the illicit use of the ergative case with the imperfective aspect marked light verb. In Hindi compound light verb constructions, the first/main verb (V1) appears in the root form and lacks grammatical markers, so it cannot independently assign a case. Thus, whether the subject case of our stimuli constitutes a correct or incorrect case assignment becomes clear solely at the position of the second/light verb (V2).

Furthermore, there are no morphological breaches present in any of the other sentential positions (NP1, NP2, V1, Aux), including the position of light verbs (V2), that would trigger any other semantic or morphological violations in the experimental conditions. In our comparison conditions, NOM-IMPF to *ERG-IMPF and ERG-PERF to *NOM-PERF, only the subject case assignment at NP1 changes visually, while everything else remains constant. Therefore, any ERP difference observed at the light verb would reflect processing differences between the two subject case assignment patterns.

We posit the following competing hypotheses for the present study. Firstly, if the processing mechanism uniformly treats ergative and nominative cases, we expect qualitatively similar ERP effects for both case violation types compared to their correct counterparts, thereby supporting a generalized case processing mechanism. Following the ERP studies in Hindi [32] and Punjabi [31] with a similar case–aspect-based violation realized on the transitive verb, we expect a P600 effect for both the violation as a marker conflict monitoring associated with identifying case-based ill-formed constructions.

Prior ERP studies on case processing from split-ergative languages have reported heterogeneous findings. In simple transitive structures (SOVs), when the critical position occurs on the main verb, Hindi elicited a P600 for ergative case–aspect violations [32], Punjabi elicited a P600 for a mismatch of both ergative and nominative case with aspect [31]. Meanwhile, another study from Hindi [29] elicited a biphasic N400-P600 for an ergative case-imperfective aspect violation and an N400 for a nominative case-perfective aspect violation. Hence, if neurophysiological differences underlie ergative and nominative case assignment, our alternative hypothesis is the expectation of distinct neural correlates, suggesting case-specific processing differences. We constructed a similar research design to the previous ERP study in Hindi [29], following a morphosyntactic violation paradigm. The present study also tested the grammatical match/mismatch of the aspect-based split-ergative case alignment pattern. The only difference between [29] and the present study is the use of a compound light verb structure, not as an object of investigation but as a novel structural environment to test case comprehension. Thus, in line with [29], if there is a processing difference between ergative and nominative case comprehension, it should evoke similar ERP effects in Hindi as [29]. We expect the case processing difference to elicit a biphasic N400-P600 effect for the ergative case-imperfective aspect violation and an N400 for the nominative case-perfective aspect violation.

A recent ERP study in Hindi [42] investigated whether the distinction in the type of light verb would evoke a difference in the processing of case. This study examined the processing of ergative versus nominative case, employing a transitivity-based split-ergative system in Hindi compound light verb constructions. The interplay of the case (ergative, nominative) and transitivity of light verbs (transitive, intransitive) was manipulated in a violation paradigm. The comparison conditions in [42] contrasted transitive versus intransitive light verb structures in each case type. This research design differs entirely from the present study, which kept the transitivity rule intact while manipulating aspect-based split-ergative alignment and compared the processing of two case types within each aspectual light verb predicate. In compound light verb structures, Hindi evoked completely distinct results in [42] compared to previous ERP studies on case processing in Hindi [29]. Therefore, we have another alternate hypothesis. If the ERP effects in the present study differ from prior case processing evidence from Hindi and other ergative languages, it would indicate that case processing varies across structures. This variation stems from the differences in split-ergative rules and structural contexts that previous studies employed to examine case and case-based violations. This would specifically distinguish between simple verbs and complex predicate structures within the same language.

## 2. Materials and Methods

### 2.1. Participants

The study recruited twenty-four native Hindi speakers (age range: 17–30 years, mean age: 25 years; 8 females and 16 males), predominantly comprising graduate and postgraduate students, as well as faculty/staff members, at the Indian Institute of Technology Ropar. The participants primarily hailed from Hindi-speaking regions, including Delhi, the National Capital Region (NCR), Uttar Pradesh, and Madhya Pradesh, and had acquired Hindi as their first language before age five. All participants had undergone formal training in the Hindi and English languages as part of their schooling. Considering India’s rich linguistic landscape, it is plausible that a substantial proportion of the participants were exposed to other regional languages, in addition to exhibiting a high level of competence in English. Nevertheless, Hindi was their dominant language.

In accordance with established research protocols approved by the institution’s Ethics Committee (Human), the participants provided informed written consent before beginning the experiment. The participant pool was further characterized by normal or corrected-to-normal vision, normal hearing, and the absence of any documented reading or neurological disorders. Additionally, the handedness assessment was conducted using an abridged version of the Edinburgh Handedness Inventory in Hindi [74], which confirmed that all participants exhibited right-handed dominance.

The final analysis excluded data from fourteen additional participants due to excessive EEG artefacts or insufficient accuracy on the behavioral task (error rate exceeding 20% in any condition).

### 2.2. Material

The experimental design included 240 critical sentences, derived from 60 sets in four conditions, following the canonical SOV word order (illustrated in Table 1). This resulted in 240 critical sentences, which were divided into two lists, each containing 120 critical sentences. The 120 critical sentences in each list means that every list contained 30 sentences for each of the four conditions. Each list containing 120 critical sentences was interspersed with 120 filler sentences. The participants were randomly assigned one of the two lists, with list assignment systematically counterbalanced to minimize potential biases. This was visually presented to the participants in a pseudo-randomized sequence.

To construct our 240 critical sentences, we utilized three transitive light verbs. Transitive light verbs were selected based on corpus frequency and established theoretical diagnostics. Specifically, high-frequency light verbs were identified from the Hindi/Urdu Treebank [75,76] and evaluated against inclusion and exclusion criteria proposed in the descriptive and theoretical literature on light verb constructions [43,54,56,77,78]. These light verbs, in their masculine and feminine forms, were repeated over twenty times across the four critical conditions. A counterbalance was achieved between the variety and frequency of aspect markers (imperfective and perfective) and gender markers (masculine and feminine) used to create light verb forms that either exhibited case–aspect rule alignment or a violation. Accordingly, a balance was also maintained in the gender of the arguments using both masculine and feminine subject and object arguments. This prevented any one type of light verb, aspect, or gender marker from always being viewed as a violation. The repetition of the light verbs was necessary because only a small subset of Hindi verb classes is permitted to function as light verbs. Moreover, even a smaller fraction of light verbs is compatible with the required experimental structural configuration. There were no additional repetitions of the lexical items in the experimental sentences. The experimental materials, including critical and filler sentences, as well as instructions, were presented visually in Hindi (Devanagari script) to participants. The study also incorporated diverse filler types into the stimuli, which effectively prevented participants from developing strategic response biases. These fillers were also balanced for the number of grammatical and ungrammatical sentences, ensuring a more robust experimental design.

### 2.3. Procedure

The experimental procedure commenced with briefing the participants about the experiment, along with providing them with a detailed printed instruction sheet. However, the specific research question under investigation was not disclosed to prevent any potential bias in the data. Upon receiving an informed written consent from the participants, they completed a comprehensive questionnaire pertaining to their linguistic background information. Additionally, they completed an adapted Hindi version of the Edinburgh Handedness Questionnaire [74]. Subsequently, the precise head measurements of the participants were obtained, and a Hydrocel GSN net of appropriate size was carefully positioned on each participant’s scalp. In a sound-attenuated experimental chamber, participants sat comfortably in a chair positioned one meter from a 20” LCD monitor displaying stimuli in a rapid serial visual presentation. Stimulus was presented using E-prime 2.0 (Psychology Software Tools, Pittsburgh, PA, United States [79]).

The experimental session began with a short practice session followed by the main experiment. A short practice session with 12 items preceded the main experiment, helping participants get accustomed to the trial format. These practice items were distinct and unrelated to the stimuli used in the subsequent experimental trials. The main experiment was divided into six blocks of 40 sentences each, with brief breaks at the end of each block. The entire experiment, including the electrode preparation, head size measurement, EEG scalp net placement, and stimulus presentation, lasted approximately two hours.

The design of each experimental trial adhered to the following structure. Each trial began with a fixation sign ‘+’ at the center of the screen for a period of 1000 ms, followed by a blank screen for a duration of 200 ms. Following the blank screen, the sentence components appeared sequentially: an adverb, followed by the first argument (NP1), then the second argument (NP2), the main verb (V1), the light verb (V2), and finally the auxiliary (Aux). The rapid serial visual presentation (RSVP) paradigm was utilized for stimulus presentation, where sentences appeared in the center of a computer screen in a phrase-by-phrase manner, with units such as determiners and nouns or nouns with case markers being presented together. Every visual chunk was presented for a duration of 650 ms with an inter-stimulus interval (ISI) of about 200 ms. This presentation time was chosen due to the orthographic and/or morphological complexity of the Hindi language and was considered a comfortable reading pace for the participants [80].

At the end of each sentence, participants performed two behavioral tasks. The first task was an acceptability judgment task. After the display of the stimulus, a visual sign ‘???’ appeared in the middle of the screen for a duration of 1500 ms, which required the participants to perform an acceptability judgment task. They had to press the green button on the response pad to indicate that the sentence presented was acceptable, or the red button to indicate that the sentence previously presented was unacceptable. After the completion of the acceptability judgment task or after a maximum of 1500 ms had passed, the participants were presented with a probe detection task, whereby a probe word appeared in the middle of the computer screen. Participants were then required to press the green button if the probe word was present in the preceding sentence, or the red button if it was not. Half of the probe words were present in the preceding trial and required a ‘yes’ response, whereas the other half were new and required a ‘no’ response. The maximal response time for the probe detection task was 3000 ms. This concluded the main experimental session.

The experimental design implemented a balanced distribution of stimuli, with an equal proportion of grammatically correct and incorrect sentences. The probe detection task was similarly counterbalanced, with an equal number of correct and incorrect probe words. Moreover, they were carefully constructed to ensure that all word categories (NP1, NP2, V1, and V2) were represented with equal probability. Furthermore, the experiment controlled for response biases by counterbalancing the assignment of ‘yes’ and ‘no’ responses to green and red buttons across participants, with half responding ‘yes’ with their left hand and ‘no’ with their right hand, and vice versa. Once the experiment was completed, the participants were asked to provide feedback and received commensurate remuneration for their participation.

### 2.4. EEG Data Acquisition and Preprocessing

The scalp EEG was recorded using the 32 Ag/AgCl electrodes (32 + VREF) fixed to the scalp with the help of a Hydrocel Geodesic Sensor Net 32 channel (Electrical Geodesics, Philips Neuro, Eugene, Oregon, United States of America). The recordings from the scalp electrodes were referenced to the vertex electrode (Cz) and re-referenced offline to the average activity of the left and right mastoids. The electrooculogram (EOG) was monitored by means of electrodes placed at the outer canthus of each eye for the horizontal eye movements and the electrodes placed above and below the eye for the vertical eye movements. All EEG and EOG channels were amplified using a NET AMPS 400 Amplifier and digitized with a down-sampling rate of 500 Hz. As per the system recommendations, the inter-electrode impedance was kept below 50 kΩ (amplifier input impedance > 1 GOhm) [81].

EEG data preprocessing was executed by exporting raw EEG data into MATLAB (version R2022b; The MathWorks, Inc.), and they were processed using EEGLAB Toolbox ([82] version 14, sccn.ucsd.edu) and the ERPLAB plugin [83]. The raw data were filtered using a bandpass filter of 0.3–30 Hz to remove slow drifts from the signal and were later re-referenced offline to the average of the left and right mastoids. A visual inspection was performed to scan and remove breaks/pauses between blocks and specific data epochs and electrodes that showed non-physiological artefacts. This is followed by the automatic rejection of the bad channels, which was performed using the pop_rejchan function in the EEGLAB toolbox. Participants with 25% or more bad channels were rejected. The data is filtered again, this time with a bandpass of 1–40 Hz. Finally, the data underwent another round of visual inspection to exclude any other types of artifacts, primarily non-physiological artifacts, such as slow drifts arising from electrical devices or wires within or around the recording environment, as well as physiological/biological artifacts, including glossokinetic and other types of body muscle movements [84]. The data was subsequently submitted for an Independent Component Analysis (ICA) [85]. In the next step, the weights calculated during the ICA step were transposed onto the file with rejected channels, and the SASICA plugin (in EEGLAB v14) was used to identify and reject all the remaining systematic artefacts, such as pulse, heartbeat, respiration, sweat, and eye movements (blink, lateral rectus spikes from lateral eye movement), as well as muscle activity artifacts [86]. Later, the missing electrodes were interpolated from the remaining channels. Baseline corrections were not performed to ensure that critical ERP epochs were not lost [29]. To ensure reliable ERP analysis, a trial retention threshold of ≥75% was applied for each critical sentence position (NP1, NP2, VP1, VP2). Participants with retention rates below this threshold at any position were excluded from the analysis. This cutoff balanced the need for sufficient trials in ERP averaging with adequate participant retention for statistical power, aligning with established ERP methodology guidelines [84]. The continuous waveform was then epoched into single trials from 200 ms before the onset of the critical position to 1200 ms relative to the onset of the critical position (light verb; −200 ms to 1200 ms). The ERPs were averaged offline for each participant at each electrode (relative to a 200 ms pre-stimulus baseline) and within each condition. In the final phase, a grand average was computed for all participants.

### 2.5. Statistical Data Analysis

For the behavioral data analysis, the mean acceptability and accuracy, as well as the mean reaction times for the acceptability judgement task and probe detection task per participant per condition, were calculated for each correct trial using E-Prime 2.0 [80]. The statistical analysis of the behavioral data was analyzed by fitting generalized linear mixed-effects models using the lme4 package [87] in R (Version 4.4.2, R Core Team, 2024) to examine the factors case at NP1 (ergative vs. nominative) and the aspect of the light verbs at VP2 (perfective vs. imperfective) for the selected participants.

For the EEG data analysis, the inclusion of EEG datasets in the final analysis was contingent upon meeting two criteria: when participants achieved at least 80% accuracy on the probe detection task and when the recordings were free from excessive EEG artifacts. The mean amplitudes in the time window of interest were statistically analyzed using the single-subject average EEG epochs at the verb for each critical condition by fitting linear mixed-effects models in R (version 4.4.3, R Core Team 2024) using the lme4 package [87]. The statistical models included the fixed factors case (ergative vs. nominative) and aspect (perfective vs. imperfective), as well as the topographical factor regions of interest (ROI). We included the mean amplitude from the 200 ms pre-stimulus period (−200 to 0 ms) as a (scaled and centered) covariate in the model for each data epoch [88]. Categorical fixed factors used sum contrasts (scaled sum contrasts for two-level factors) so that the coefficients represent deviations from the grand mean [89].

**Figure 1:**
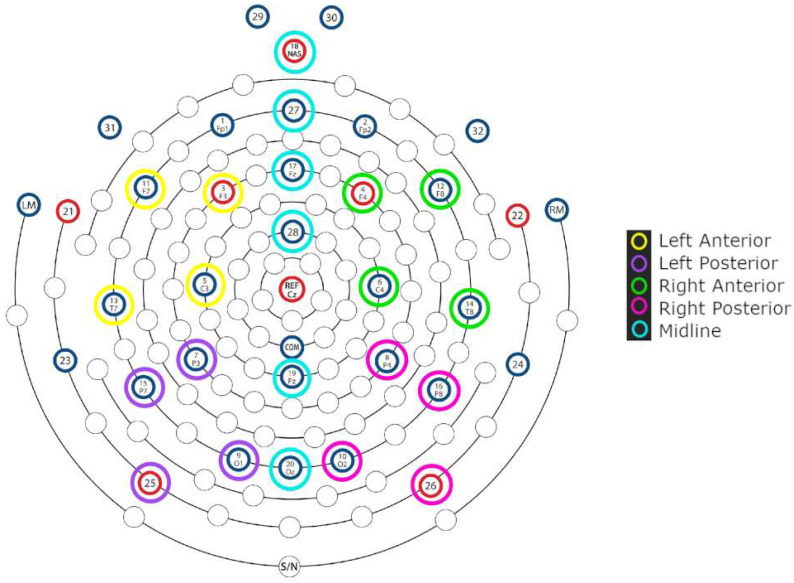
Electrode grouping of selected regions of interest for statistical analysis (adapted from Hydrocel Geodesic Sensor Net 32 Channel montage by Magstim, EGI). EEG recordings were referenced online to the vertex electrode (Cz) and re-referenced offline to the average of the left and right mastoid electrodes (LM, RM).

The ROIs were defined by clustering topographically adjacent electrodes in 4 lateral and 6 midline regions. The lateral ROIs were as follows: left anterior (3, 5, 11, 13), left posterior (7, 9, 15, 25), right anterior (4, 6, 12, 14), and right posterior (8, 10, 16, 26). For the midline region, each electrode was analyzed as an ROI of its own: 18, 27, 17, 28, 19, and 20. Figure 1 illustrates the lateral and midline sites, which underwent separate statistical analyses [90].

## 3. Results

### 3.1. Behavioral Results

**Figure 2:**
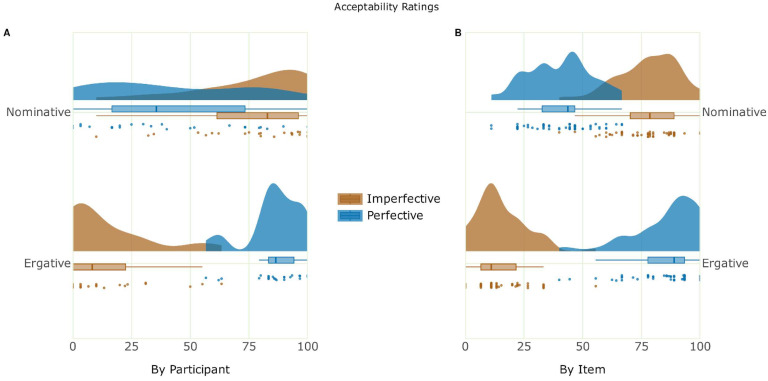
Raincloud plot of the acceptability ratings. (**A**) The by-participant variability of acceptability ratings, with the individual data points representing the mean by-participant acceptability of each case and aspect combination. (**B**) The by-item variability of acceptability ratings, with the individual data points representing the mean by-item acceptability of each case and aspect combination.

The mean acceptability ratings and probe detection accuracy for the critical conditions are shown in Table 2. The behavioral results showcase a close association between acceptability and grammaticality, as the ungrammatical conditions (*ERG-IMPF, *NOM-PERF) showed a significant decline in acceptability ratings compared to their grammatical counterparts (ERG-PERF, NOM-IMPF). The findings indicate that Hindi speakers find ungrammatical conditions with ergative case violations highly unacceptable, consistent with the language’s aspectual split-ergativity rules. This suggests that speakers are sensitive to the marked nature of ergative case assignment, which is structurally conditioned, as opposed to the default nominative case assignment. The mean probe detection accuracy was high and relatively stable across all four conditions. Figure 2 showcases the raincloud plot [91] of the acceptability judgement task.

The behavioral data were analyzed by generalized fitting linear mixed-effects models using the lme4 package [87] in R to examine the relationship of sentence acceptability and accuracy to the independent variables, case and aspect. The contrasts for the categorical variables used sum contrasts (scaled sum contrasts for two-level factors), such that coefficients reflect differences from the grand mean [91]. Model selection was based on the Akaike Information Criterion (AIC), whereby the model with the lowest AIC was selected as the one that best explained the data. The analysis of the acceptability data showed that the model involving the factors case, aspect, and their interaction terms, with random intercepts for participants and items, and by-participant random slopes for the effect of case, aspect, and their interaction terms, and by-item random slopes for the effect of aspect, best explained the acceptability data (AIC = 2331.4). Models involving more complex/no random slope specifications were either singular or had a higher AIC. Type II Wald chi-square tests on the selected model showed main effects of case (χ^2^(1) = 5.88, *p* = 0.01) and aspect (χ^2^(1) = 12.35, *p* < 0.001), as well as an interaction between case x aspect (χ^2^(1) = 131.05, *p* < 0.001). Estimated marginal means on the response scale were computed to resolve this interaction, which revealed that there was a simple effect of aspect both when the case was ergative (estimate = −0.811, SE = 0.0359, LCL = −0.860, UCL = −0.761, *p* < 0.001) and when it was nominative case (estimate = 0.481, SE = 0.098, LCL = 0.347, UCL = 0.616, *p* < 0.001). The analysis of the accuracy of the probe detection task detected no effects, reflecting the high accuracy that was similar (ceiling) across conditions.

### 3.2. Electrophysiological Results

**Figure 3:**
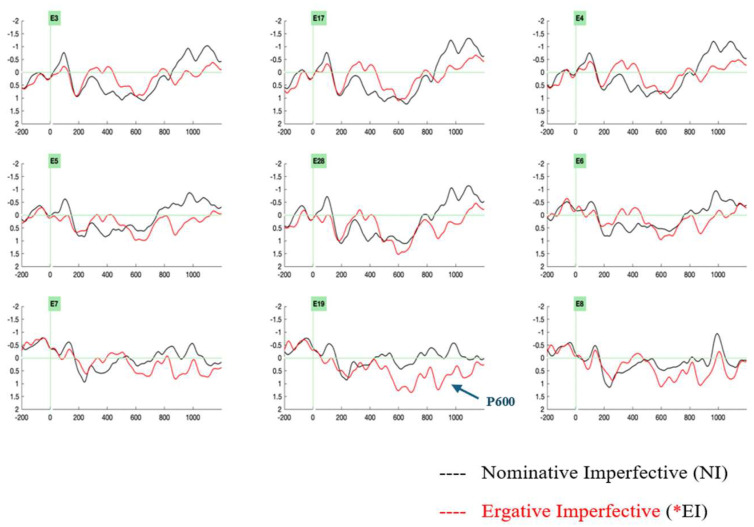
Grand-averaged ERPs from 24 participants at the light verb position comparing the control condition, nominative–imperfective aspect (marked in black), and the critical violation condition, ergative–imperfective aspect (marked in red), reveal a late positivity effect for the ergative–imperfective aspect violation condition (*EI). Negativity is plotted upwards. Abbreviations: left anterior electrodes (3, 5), midline electrodes (17, 28, 19), right anterior electrodes (4, 6), left posterior electrodes (7), right posterior electrodes (8).

**Figure 4:**
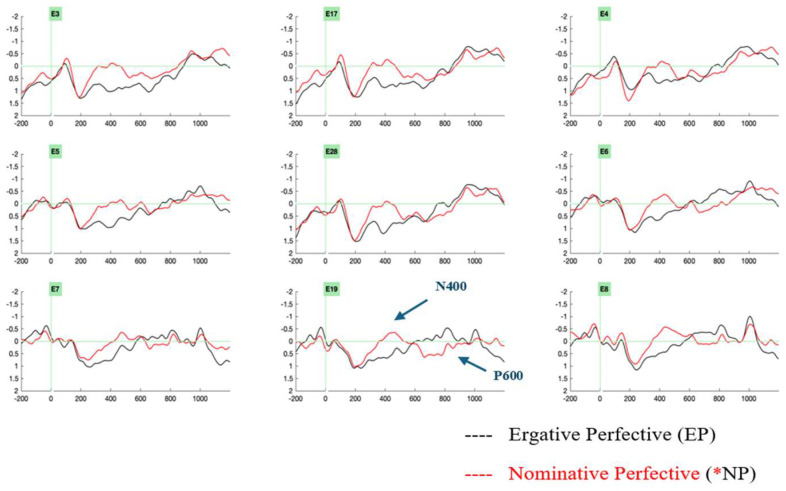
Grand-averaged ERPs from 24 participants at the light verb position comparing the control condition, ergative–perfective aspect (marked in black), and the critical violation condition, nominative–perfective aspect (marked in red), reveal a negativity–positivity effect for the nominative–perfective aspect violation condition (*NP). Negativity is plotted upwards. Abbreviations: left anterior electrodes (3, 5), midline electrodes (17, 28, 19), right anterior electrodes (4, 6), left posterior electrodes (7), right posterior electrodes (8).

The ERPs were analyzed at the critical position of the light verb. Visual inspection of the ERP data revealed two ERP effects. The ergative case-imperfective aspect violation condition (*ERG-IMPF) revealed a late positivity in the 650–850 ms time window (Figure 3). A biphasic negativity–positivity effect ensued for the nominative case-perfective aspect violation condition (*NOM-PERF), wherein negativity was elicited in a 350–550 ms time window and late positivity was elicited in a 650–850 ms time window (Figure 4).

The ERP data were analyzed by fitting linear mixed-effects models using the lme4 package in R to examine the relationship of mean ERP amplitude to the factors case (ergative, nominative) and aspect (imperfective, perfective), as well as the topographical factor region of interest (ROI), and their interaction. Model selection was based on the conditional Akaike Information Criterion (cAIC) computed using the cAIC4 package [92], whereby the model with the lowest cAIC was selected as the one that best explained the data. Estimated marginal means using the emmeans package [93] were computed on the fitted model to resolve interactions. Categorical factors used scaled sum contrasts (i.e., coefficients reflect differences from the grand mean). Instead of performing a traditional baseline correction, the mean prestimulus EEG amplitude (−200 to 0 ms) was included in the model as a scaled and centered covariate [88]. However, we do not interpret effects involving prestimulus amplitudes, in line with the fact that these did not form part of our hypotheses but also because they were included in the model to account for and regress out potential contributions of the baseline period to the critical period. Instead, we focus on effects attributable to the experimental factors case and aspect, and for each model, we interpret the highest-order interaction involving both factors. Additional interactions with the prestimulus interval are, nevertheless, resolved for a range of prestimulus amplitude values from −5 to 5 μV, which showed that the overall pattern of results remained broadly consistent (see the data repository online, which also includes the ERP analyses at the positions of NP1 and V1 for reference).

#### 3.2.1. The ERP Time Window: 350–550 ms

The analysis in this time window in the lateral regions of interest showed that the model involving the factors ROI, case, aspect, and their interaction terms, with random intercepts for participants, and by-participant random slopes for the effect of case, aspect, and their interaction terms, best explained the mean ERP amplitude (cAIC = 960.68). Models involving less complex/no random slope specifications either did not converge or had a higher cAIC. Type II Wald chi-square tests on the selected model showed an effect of the interaction between case x aspect (χ^2^(1) = 4.87, *p* = 0.02). Resolving this by computing estimated marginal means revealed that there was a simple effect of case (estimate = −0.610, SE = 0.262, LCL = −0.980, UCL = −0.239, *p* = 0.02) when the aspect was perfective.

The analysis in the midline regions of interest showed that the model involving the factors ROI, case, and aspect, and their interaction terms, with random intercepts for participants and by-participant random slopes for the effect of case, aspect, and their interaction terms, best explained the mean ERP amplitude (cAIC = 1957.30). Models involving less complex/no random slope specifications either did not converge or had a higher cAIC. Type II Wald chi-square tests on the selected model showed an effect of the interaction between aase x aspect (χ^2^(1) = 3.68, *p* = 0.05). Resolving this by computing estimated marginal means showed a simple effect of case (estimate = −0.582, SE = 0.298, LCL = −1.003, UCL = −0.161, *p* = 0.06) when the aspect was perfective.

#### 3.2.2. The ERP Time Window: 650–850 ms

The analysis in this time window in the lateral regions of interest showed that the model involving the factors ROI, case, aspect, and their interaction terms, with random intercepts for participants, and by-participant random slopes for the effect of case, aspect, and their interaction terms, best explained the mean ERP amplitude (cAIC = 899.71). Models involving less complex/no random slope specifications either did not converge or had a higher cAIC. Type II Wald chi-square tests on the selected model showed an effect of the interactions ROI x aspect (χ^2^(3) = 6.64, *p* = 0.08) and ROI x case x aspect (χ^2^(3) = 6.77, *p* = 0.07). Resolving these by computing estimated marginal means detected no further effects.

The analysis in the midline regions of interest showed that the model involving the factors ROI, case, aspect, and their interaction terms, with random intercepts for participants and by-participant random slopes for the effect of case, aspect, and their interaction terms, best explained the mean ERP amplitude (cAIC = 2052.64). Models involving less complex/no random slope specifications either did not converge or had a higher cAIC. Type II Wald chi-square tests on the selected model showed an interaction effect of ROI x case x aspect (χ^2^(5) = 37.00, *p* < 0.001). Estimated marginal means computed to resolve this interaction showed that there was a simple effect of case in ROI = M1 (estimate = 1.093, SE = 0.541, LCL = 0.345, UCL = 1.842, *p* = 0.04) and ROI = M5 (estimate = −1.09, SE = 0.538, LCL = −1.840, UCL = −0.351, *p* = 0.04) when the aspect was imperfective. Further, there was a simple effect of case in ROI = M5 (estimate = 1.101, SE = 0.503, LCL = 0.406, UCL = 1.797, *p* = 0.03) when the aspect was perfective.

## 4. Discussion

The present ERP study examined the processing case in Hindi compound light verb constructions. The study employed an aspect-based split-ergativity system to investigate whether the interplay of case (ergative, nominative) and aspect (imperfective, perfective) would evoke similar or distinct neural correlates. The electrophysiological results at the position of the light verb showed that an ergative case violation at the imperfective aspect marked light verb evoked a late positivity effect in the 650–850 ms time range, in contrast to its grammatical counterparts. On the other hand, the nominative case violations realized on the perfective aspect marked light verb elicited a biphasic effect with negativity in the time range of 350–550 ms and a late positivity in the time range of 650–850 ms, in contrast to its grammatical counterparts. These effects can be plausibly interpreted as instances of a P600 effect and an N400-P600 biphasic effect, respectively. Therefore, our electrophysiological results reveal different neural correlates for the two types of subject case assignments in Hindi light verb structures, namely, ergative versus nominative case assignments.

The present study solely tested the grammatical requirement of the case–aspect-based split-ergativity rule, creating either correct or illicit ergative and nominative case-based compound light verb constructions. As previously pointed out in Hindi’s typological Section 1.1, light verb structures exhibit a complex encoding of syntactic and semantic properties wherein the argument structure and event meaning are co-composed within a universal predicational framework. It was beyond the investigatory capacity of the present study to simultaneously examine the processing of different types of predicate structures [34,38] or study the inherent semantic inferences for event culmination in simple predicates [94] versus light verb predicate structures. This is an avenue future studies can explore.

Further, upon comparing the results from the present study, which examined the case and aspect alignment mismatch in compound light verb constructions, to the previous electrophysiological findings from ergative languages, we can observe contradictory evidence for case processing. In Basque, [30] observed a similar ERP pattern in transitive structures (OVS) to that previously reported by [29] in Hindi, namely, an N400 for nominative case violations and an N400-P600 effect for ergative case violations. However, another ERP study in Basque [27] reported a P600 effect for double ergative case violations, contradicting [30]’s results. In Hindi, [29,32] found distinct ERP patterns for ergative case–aspect mismatches realized on simple transitive verbs, with the former study reporting a biphasic N400-P600 effect and the latter a RAN-P600 effect. In Punjabi, ref. [31] reported an early positivity for the nominative case violations and a late positivity for the ergative case violations. Following a similar morphosyntactic violation paradigm from these previous ERP studies on case processing from ergative languages, the present study constructed a similar experimental design. Specifically expanding on the ERP studies from Hindi [29] and Punjabi [31], the present study constructed a grammatical match/mismatch of the aspect-based split-ergative case alignment pattern. But, in contrast to earlier studies [29,31,32], the present study utilized compound light verb constructions rather than simple transitive structures. Our finding, in line with a previous ERP study in Hindi [29], provides converging support to the fact that the nominative and ergative cases are processed differently. However, the ERP components observed are not the same. For instance, we expected to see only an N400 for the nominative–perfective violations and an N400-P600 for the ergative–imperfective violations, in line with [29], as both studies exploited the Hindi case and aspect alignment mismatch. Nevertheless, our ERP findings from the present study evoked a distinct ERP pattern for the processing of ergative versus nominative case assignment in Hindi compound light verb structures.

In the present study, we avoided comparing perfective and imperfective aspect-marked light verbs within each case type. Such a comparison could create differences in types of verbs, acting as a confound and obscuring our main goal of understanding case comprehension. However, extending the research question to involve different types of light verbs and studying their impact on case comprehension remains a potential direction for future research. Recently, an ERP study on case processing from Hindi [42] attempted to address a research question along these lines. This study employed compound light verb constructions to examine the processing of case, utilizing the transitivity-based split-ergativity system. The authors reported an N400 for ergative case-intransitive light verb violations and a P600 effect for the nominative case-transitive light verb violations. The results from [42] further contradict those of the present study, which examined the processing of case and case-based violations while manipulating the aspect-based split-ergativity system. All of these findings collectively confirm that there are qualitative differences between processing nominative and ergative cases across different ergative languages that may display various ergativity patterns. Furthermore, these differences persist within a single ergative language, manifesting across diverse sentential structures and multiple cues as they interact within a particular sentence structure for sentence comprehension.

In the following sections, we outline possible interpretations for the N400 and P600 ERP components observed in the present study and discuss their potential implications for future research. In general, the N400 and P600 ERP effects observed in this study can be interpreted by several existing accounts. We interpret the N400 effect evoked for the nominative case-perfective aspect violation in line with [29] as an interpretatively relevant case-based rule violation, wherein the incorrect usage of morphosyntactic rules leads to a disruption of the predicted sentence interpretation. A nominative subject (as in the *NOM-PERF condition) predicts either a verb in the imperfective aspect or an intransitive main verb to complete the unfolding sentence. However, when the perfective aspect-marked transitive light verb is encountered, it fails to fit the nominative subject case alignment. This leads to a case-based processing conflict at the light verb position, which breaks down the previously predicted interpretation, resulting in the nominative case violation eliciting an N400 effect. Furthermore, we interpret the P600 effect elicited by both the case violation conditions (*ERG-IMPF and *NOM-PERF) at the transitive light verb as a marker of the conflict monitoring mechanism [4,95], which is evoked when detecting the ill-formedness of the erroneous case-marked construction [1,29].

We propose a potential explanation for the unique findings below, specifically focusing on the absence of a negativity effect and the presence of a monophasic late-positivity effect observed in response to ergative case-imperfective aspect violations. The ERP findings from the present study illustrate how variation in cue strength and cue reliability affects incremental sentence comprehension in real-time [2,96]. The ergative case of the subject in the ergative violation conditions (*ERG-IMPF) is interpreted as an agentive argument, which leads to a strong expectation for a transitive–perfective event structure to unfold. When a transitive polar verb (V1) is encountered, the expectation of a transitive predicate structure that the ergative subject generated is fulfilled. When the imperfective light verb (V2) is subsequently encountered, it does not appear to hinder the interpretation of the core meaning of the event. In other words, although a transitive imperfective light verb predicate requires the subject to be in the nominative case, the overall comprehension of the event itself proceeds unhindered, even if the subject was superfluously marked ergative, as in the case of the *ERG-IMPF conditions (which is prescriptively ungrammatical in Hindi). That is, the predicate information appears to be a reliable enough cue to interpret the event rather than the subject case, and the anomalous ergative case of the subject could be ignored as redundant as far as arriving at a meaningful interpretation of the event based on the transitive–imperfective predicate is concerned, even though the anomaly is detected and categorized as ill-formed, thus engendering a P600 effect. Ignoring the superfluous ergative marking of the preceding subject argument appears to be less costly, which could explain the absence of an N400 effect for ergative case-imperfective aspect violations. By contrast, in the nominative violation conditions (*NOM-PERF), even though the nominative subject can entail diverse sentence structures, it cannot function as the explicit ergative agent that is obligatorily required by the transitive–perfective predicate. When this interpretively relevant linguistic rule that requires an ergative agent for a transitive–perfective event is violated, it hinders interpreting the event correctly, and an N400 ensues [29]. In other words, unlike a superfluous ergative marking that could be ignored without concomitant costs, a required ergative marking that is clearly absent from the input cannot be recreated in the internal model. 

Our findings align with the cue validity framework [2,3] and the conflict reliability model [4], suggesting that cue strength depends on how native speakers weigh and employ multiple cues simultaneously in language processing. The experimental evidence from the present study highlights how the case, as a processing cue, is prone to neurophysiological variation when interacting with other processing cues within light verb structures. However, more follow-up studies are required to disentangle differences in cue type and cue necessity. This is because the relative strength of cues can vary across languages and structures, influencing comprehension. Furthermore, the relationship between sentence structure and event comprehension is complex, and it is particularly evident in compound light verb structures, where the intricate mapping of ergativity and transitivity patterns significantly influences the processing of case markers, argument prediction, and event construction. These complex predicate structures diverge substantially from their simple transitive counterparts, necessitating a deeper examination of the cognitive mechanisms underlying language processing. Our study sheds light on this, revealing that in Hindi, perfectivity and transitivity emerge as robust cues for case assignment, even in the context of light verb structures.

## 5. Conclusions

To conclude, our findings of different ERP correlates, namely, the N400-P600 effect for nominative–perfective violations and the P600 effect for ergative–imperfective violations, differ from earlier findings on case processing in ergative languages. This suggests that the processing mechanism exhibits structure-specific neurophysiological differences. It also underlines the complexity of case processing mechanisms, suggesting against overgeneralizing findings across languages. Therefore, the present study highlights neurophysiological variations both within and across ergative languages, reinforcing the notion that these languages exhibit cross-linguistic variability. Although further research on understudied languages and structures is evidently needed, this study significantly showcases how structural variation influences case comprehension.

## Figures and Tables

**Table 1:** Experimental sample.

Condition	Sample Sentences					
(a.) NOM-IMPF	लेखक		पत्र	पढ़	लेता	है ।
	Lekhak		patr	padh	le-taa	hai
	Writer.3SG.M.NOM letter.3SG.M.NOM read				take.IMPF.M.3SG.LV	Aux.SG.PRS
	‘The writer reads the letter’					
(b.) *ERG-IMPF	लेखक	ने	पत्र	पढ़	लेता	है ।
	Lekhak-ne		patr	padh	le-taa	hai
	Writer.3SG.M-ERG letter.3SG.M.NOM			read	take.IMPF.M.3SG.LV	Aux.SG.PRS
	‘The writer reads the letter (intended)’					
(c.) *NOM-PERF	लेखक		पत्र	पढ़	लिया	है ।
	Lekhak		patr	padh	li-yaa	hai
	Writer.3SG.M.NOM letter.3SG.M.NOM			read	take.PERF.M.3SG.LV	Aux.SG.PRS
	‘The writer has read the letter (intended)’					
(d.) ERG-PERF	लेखक	ने	पत्र	पढ़	लिया	है ।
	Lekhak-ne		patr	padh	li-yaa	hai
	Writer.3SG.M-ERG letter.3SG.M.NOM			read	take.PERF.M.3SG.LV	Aux.SG.PRS
	‘The writer has read the letter’					

**Legend**: The ERPs were time-locked to the critical position of the light verbs, and the light verbs wherein the violation occur are highlighted in red. The use of an asterisk ‘*’ indicates a violation of the case assignment rule, resulting in a mismatch between the case of the subject argument and the aspect markers on the light verb. Abbreviations: ERG: ergative case; NOM: nominative case; M: masculine; F: feminine; SG: singular; 3: third person, PERF: perfective aspect of the light verb; IMPF: imperfective aspect of the light verb; LV: light verb; Aux: auxiliary; PRS: present tense.

**Table 2:** Mean acceptability ratings and probe detection accuracy.

Condition	Acceptability Judgment Task		Probe Detection Task	
	Acceptability (%)	RT 1 (ms)	Accuracy (%)	RT 2 (ms)
(a.) NOM-IMPF	76.7 (42.3)	461 (142)	96.3 (18.9)	650 (102)
(b.) *ERG-IMPF	14.9 (35.6)	437 (150)	94.5 (22.8)	691 (122)
(c.) *NOM-PERF	41.6 (49.3)	382 (70)	95.6 (20.5)	680 (108)
(d.) ERG-PERF	86.0 (34.7)	442 (108)	95.7 (20.3)	649 (100)

**Legend**: Case markers are indicated as ERG, ergative case; NOM, nominative case; and aspect on the light verbs as IMPF, imperfective, and PERF, perfective. NOM-IMPF and ERG-PERF are grammatically correct sentences, and *NOM-PERF and *ERG-IMPF are ungrammatical sentences. The values represent the mean acceptability and accuracy and the respective reaction times for the two behavioral tasks per four conditions, with the standard deviations provided in parentheses (values have been rounded off).

## Data Availability

The datasets generated/analyzed for this study can be found in the following repository: https://doi.org/10.5281/zenodo.17404425.

## Abbreviations

The following abbreviations are used in this manuscript:

MDPI	Multidisciplinary Digital Publishing Institute
DOAJ	Directory of Open Access Journals
EEG	Electroencephalography
ERP	Event-Related Potential
N400	ERP Component: Negativity peaking around 400 milliseconds after a stimulus
P600	ERP Component: Positivity peaking around 600 milliseconds after a stimulus
RAN	ERP Component: Right Anterior Negativity
SG	Singular
M	Masculine
F	Feminine
ERG	Ergative Case
NOM	Nominative Case
PVF/PERF	Perfective Aspect
IMPF	Imperfective Aspect
V1	Polar Verb
LV/V2	Light Verb
LVC	Light Verb Construction
AUX	Auxiliary
PRS	Present Tense
cAIC	Conditional Akaike Information Criterion
ROI	Region of Interest
